# Artificial intelligence–based analysis of retinal vascular changes in the preclinical and early stages of diabetic retinopathy using ultra-widefield fundus imaging: an observational cross-sectional study

**DOI:** 10.3389/fmed.2026.1906162

**Published:** 2026-07-14

**Authors:** Tingting Sun, Yingjun Min, Caihong Wang, Nurbiya Abduriyim, Chenting Wen, Qinghua Qiu

**Affiliations:** 1 Tongren Hospital, Shanghai Jiao Tong University School of Medicine, Shanghai, China; 2 Bachu County Hospital of Traditional Chinese Medicine, Kashgar, China; 3 Department of Ophthalmology, Shigatse People’s Hospital, Shigatse, China

**Keywords:** artificial intelligence, diabetic retinopathy, retinal vascular parameters, type 2 diabetes mellitus, ultra-widefield fundus photography

## Abstract

**Background:**

Subclinical retinal microvascular remodeling may occur before clinically detectable diabetic retinopathy (DR). This study investigated artificial intelligence (AI)-derived ultra-widefield retinal vascular metrics in patients with type 2 diabetes mellitus (T2DM) with and without non-proliferative DR (NPDR), and evaluated their potential for early vascular phenotyping and diagnostic discrimination.

**Methods:**

In this observational cross-sectional single-center study, 237 participants were included: 63 healthy controls (103 eyes), 101 patients with T2DM without DR (No-DR; 201 eyes), and 73 patients with NPDR (132 eyes). Non-mydriatic 200-degree ultra-widefield fundus images were analyzed using an AI-based vascular segmentation and quantification system. AI-exported values coded as -1 were treated as missing, and sparse parameters were excluded from primary inference. Intergroup comparisons of retained vascular parameters were performed using age- and sex-adjusted mixed-effects models with participant as a random intercept, followed by Benjamini-Hochberg false discovery rate (FDR) correction. Multiparameter logistic models were evaluated using 5-fold subject-level cross-validation.

**Results:**

After quality control, covariate adjustment, and FDR correction, 38 vascular-parameter rows remained significant. Whole-field vessel density was highest in the No-DR group, intermediate in NPDR, and lowest in controls [control, 0.017 (0.008–0.023); No-DR, 0.026 (0.020–0.032); NPDR, 0.021 (0.015–0.026); FDR *P* < 0.001]. Fractal-dimension metrics showed similar early alterations, with arterial fractal dimension increased in No-DR and intermediate in NPDR [control, 1.287 (1.164–1.359); No-DR, 1.380 (1.328–1.420); NPDR, 1.329 (1.271–1.374); FDR *P* < 0.001]. Whole-field mean vessel diameter was lower in No-DR than in controls, whereas total vessel length decreased across controls, No-DR, and NPDR (FDR P < 0.001 for both). Regional heatmaps showed that significant density and fractal-dimension signals clustered mainly in the superotemporal, superonasal, and inferotemporal regions. Cross-validated models showed good discrimination for No-DR versus control (AUC, 0.853; 95% CI, 0.807–0.893), NPDR versus control (AUC, 0.785; 95% CI, 0.720–0.841), and No-DR/NPDR versus control (AUC, 0.830; 95% CI, 0.788–0.873), but more modest discrimination between NPDR and No-DR (AUC, 0.658; 95% CI, 0.596–0.720).

**Conclusion:**

AI-derived ultra-widefield retinal vascular metrics demonstrate early, spatially heterogeneous microvascular remodeling in T2DM before clinically apparent DR. Vessel density, fractal dimension, vessel diameter, and vessel length provide complementary information, and multiparameter vascular modeling may support early detection and risk stratification. External validation and longitudinal studies are required before clinical implementation.

## 1 Introduction

Diabetic retinopathy (DR) is a leading cause of preventable visual impairment among working-age adults worldwide, with its prevalence projected to increase substantially in parallel with the expanding global diabetes burden (1, 2). Although vision-threatening complications such as proliferative diabetic retinopathy (PDR) and diabetic macular edema (DME) account for most DR-related visual loss, converging evidence indicates that neurovascular dysfunction begins years before conventional clinical signs become apparent (3, 4). Subtle alterations—including early vascular caliber changes, increased tortuosity, capillary rarefaction, and peripheral perfusion deficits—may occur during the preclinical or early stages of DR but remain challenging to detect through routine clinical examination (4–6). Identifying these early microvascular phenotypes is clinically important because timely intervention can delay, or even prevent, progression to advanced stages associated with irreversible vision loss (2, 5).

Artificial intelligence (AI) has emerged as a powerful tool for characterizing retinal pathology (7–9). Deep learning systems demonstrate expert-level accuracy for DR detection, severity grading, and lesion identification across diverse imaging platforms (10, 11). Beyond classification tasks, AI-enabled vascular analytics now permit automated quantification of vessel density, fractal dimension, tortuosity, arteriovenous ratio, and other morphological biomarkers that may sensitively reflect early microvascular impairment (12, 13). These advanced quantitative metrics hold promise for improving early disease detection, risk stratification, and mechanistic understanding of DR pathophysiology.

At the same time, widefield and ultra-widefield (UWF) fundus imaging have expanded the ability to visualize the retinal periphery—an anatomical region increasingly recognized as critical in early DR (14–16). Compared with conventional 30–55° imaging (17), UWF systems can capture up to 200° of the retina in a single acquisition, enabling comprehensive assessment of peripheral nonperfusion, microvascular dropout, and regional ischemia that are often missed on standard imaging (15, 16, 18). Integrating UWF imaging with AI-driven vascular analysis offers a unique opportunity to detect subtle phenotypic changes across a substantially broader retinal area, particularly during preclinical or early disease stages in which conventional grading systems lack sensitivity.

Given these gaps, the present observational cross-sectional study evaluates AI-derived retinal vascular parameters from widefield fundus imaging in individuals with preclinical or early-stage DR. By characterizing quantitative vascular alterations associated with early disease, this study aims to advance the development of imaging-based biomarkers for early detection, risk stratification, and improved understanding of early DR pathogenesis.

## 2 Materials and methods

### 2.1 Study design

This was an observational, cross-sectional, single-center study that evaluated retinal vascular changes in the preclinical and early stages of diabetic retinopathy using artificial intelligence–based automated analysis of widefield fundus images, with consecutively enrolled participants divided into three groups: healthy control group, no-DR group (type 2 diabetes without diabetic retinopathy), and early DR group (mild non-proliferative diabetic retinopathy), conducted at Shanghai Jiao Tong University Affiliated Tongren Hospital, Shanghai, China between June 2024 and December 2025. The study was conducted in strict accordance with the principles of the Declaration of Helsinki and was approved by the Ethics Committee of Shanghai Jiao Tong University Affiliated Tongren Hospital (Approval No. K2026-057-01). All participants provided written informed consent prior to enrollment.

### 2.2 Study participants

The present study was an observational cross-sectional study. Sample size calculation was performed using IBM SPSS Statistics (Version 31.0, IBM Corporation, Armonk, NY, United States) based on the one-way analysis of variance (ANOVA) design. The primary endpoint for sample size estimation was retinal vessel density, a core morphological parameter of retinal microvasculature. Referring to previously published studies on retinal vascular alterations in preclinical and early diabetic retinopathy, the effect size was set at Cohen’s *f* = 1.00, with a two-sided significance level of α = 0.05 and a target statistical power of 90% (1–β = 0.90) for three-group comparison. The calculation indicated that a total of 18 participants (6 per group) would achieve an actual power of 0.938, exceeding the pre-specified power requirement. Taking into account a 15% dropout rate caused by unqualified fundus images, poor subject cooperation, and incomplete clinical data, we planned to enroll at least 26 participants per group to ensure adequate statistical power for all planned analyses.

### 2.3 Inclusion criteria

(1) Aged ≥ 18 years; (2) For diabetic patients: Diagnosis of type 2 diabetes confirmed by medical records; (3) Ability to complete Ultra-widefield fundus photography; (4) Provision of written informed consent.

### 2.4 Exclusion criteria

(1) Other retinal diseases (e.g., age-related macular degeneration, retinal vein occlusion, primary or secondary glaucoma); (2) Ocular media opacity affecting image quality (e.g., severe cataract, vitreous hemorrhage); (3) History of ocular surgery within 6 months prior to enrollment (e.g., cataract surgery, retinal laser photocoagulation); (4) Systemic diseases severely affecting retinal vasculature (e.g., sickle cell disease, systemic lupus erythematosus, pregnancy or lactation); (5) Inability to cooperate with image acquisition or follow-up.

### 2.5 Image acquisition and preprocessing

Ultra-widefield (UWF) fundus photographs covering a 200° field of view were acquired using a Daytona P200T ultra-widefield laser scanning ophthalmoscope (Optos PLC, Dunfermline, United Kingdom). All imaging procedures were performed under non-mydriatic conditions without administration of any pupil-dilating agents. Image collection was uniformly completed by professionally trained ophthalmic technicians. Subsequent rigorous quality control screening was implemented: images suffering from motion artifacts, uneven illumination, blurring or indistinct retinal vascular structures were excluded, and eligible participants were scheduled for re-imaging when needed.

**FIGURE 1:**
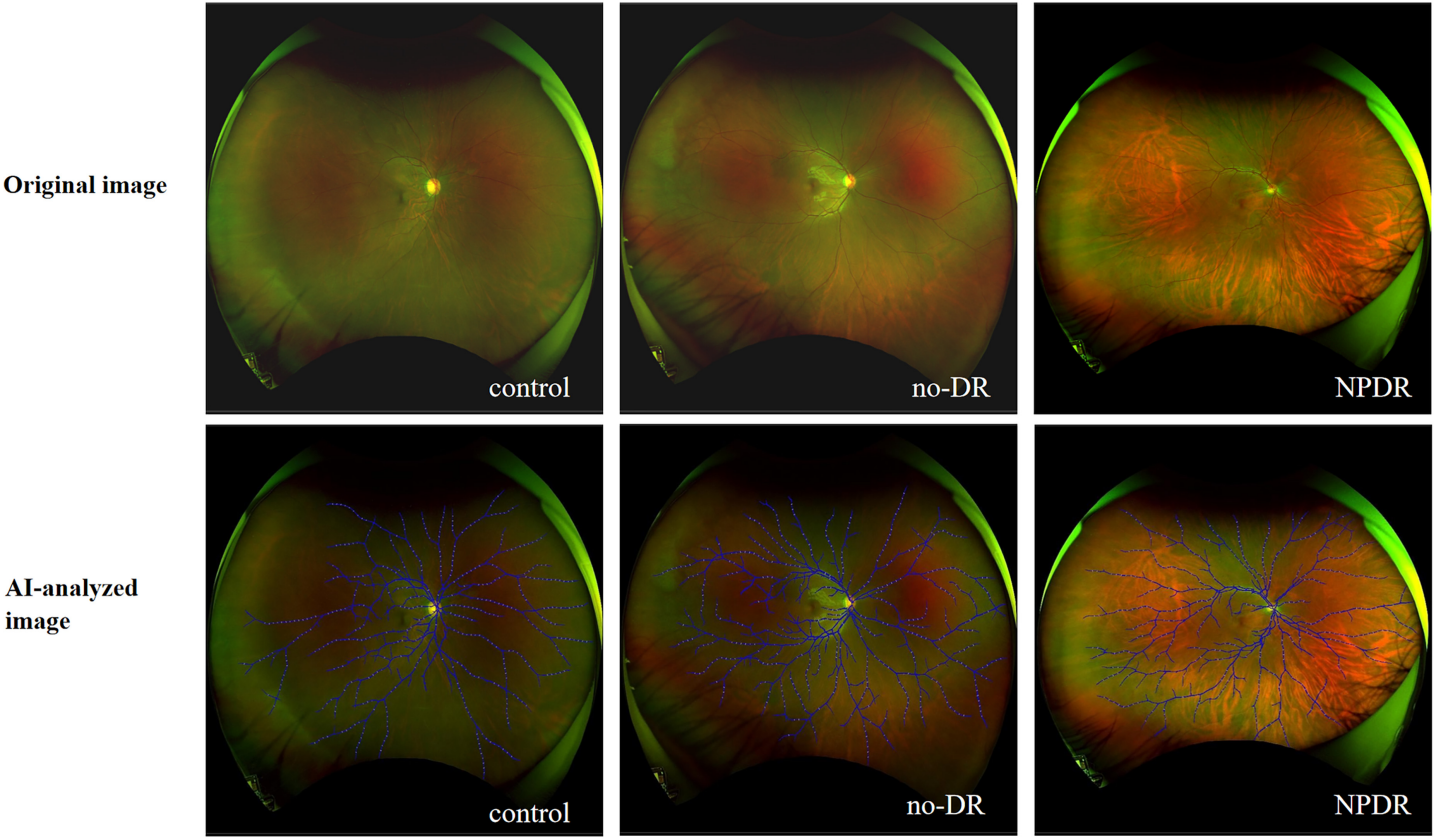
Representative ultra-widefield fundus images and corresponding AI-based retinal vascular segmentation results. The top row shows original ultra-widefield fundus images from the control group (left), no-DR group (middle), and mild NPDR group (right). The bottom row shows the corresponding AI-analyzed images, with blue lines indicating the automatically segmented retinal vascular network.

All qualified UWF fundus images were imported into the EVisionAI intelligent fundus image analysis system (Evision Technology (Beijing) Co., Ltd., Beijing, China) for automated retinal vascular segmentation and quantitative analysis (Figure 1). This platform was constructed based on a multi-stage deep learning framework inspired by human visual bionic principles, and its reliability in automated quantitative analysis of retinal vascular phenotypes has been validated in previous clinical studies. Prior to vascular feature extraction, all images underwent standardized preprocessing to unify image quality and analytical consistency. The workflow included region of interest extraction to remove redundant peripheral non-retinal interfering backgrounds, image normalization to standardize color tone, brightness and exposure levels across all samples, and Contrast Limited Adaptive Histogram Equalization (CLAHE) to enhance local contrast and sharpen edge contours of retinal vessels. Multi-channel spectral feature separation was also performed to enrich valid vascular feature information and support high-precision subsequent segmentation.

The core vessel segmentation module adopted an improved TransUnet network integrating convolutional encoder-decoder architecture and Transformer module, which is capable of capturing both subtle local microvascular morphological details and global topological distribution features of the entire UWF retinal vascular network. The encoder achieved hierarchical feature compression via convolutional layers; after linear projection and position encoding, extracted features were imported into the Transformer block to identify long-range spatial correlations of vascular distribution. The decoder restored original spatial resolution through transposed convolution, and retained fine tiny vessel features via skip connection multi-scale feature fusion. The deep learning model was trained and optimized on large-scale multicenter manually annotated fundus image datasets including the Beijing Eye Study cohort. All annotations focusing on retinal vessels and macular structures were finished by experienced ophthalmologists and further refined via semi-automatic calibration combined with manual revision. Furthermore, a visual attention mechanism-based ResNet101-UNet edge optimization algorithm was applied to refine vessel boundaries, and automatic arteriovenous differentiation was realized according to inherent differences in vascular color, brightness, texture and topological connectivity.

The favorable segmentation and analytical performance of this AI system have been fully verified in prior researches. The system achieved an AUC above 0.98 for fundus lesion assessment with sensitivity and specificity over 94%. The intersection over union (IoU) reached 0.939 for optic disc segmentation and exceeded 0.99 for retinal vessel segmentation, while the overall optic disc detection accuracy was up to 0.998. Equipped with an embedded automatic quality control module, the system can independently evaluate image clarity, illumination uniformity and artifact interference, and automatically eliminate substandard images to guarantee the credibility of vascular quantitative results.

Following automated generation of binary vessel segmentation masks and vascular centerline skeletons, the system adopted the optic disc as a unified reference coordinate system to automatically calculate multiple retinal vascular morphological parameters. Quantitative measurements were performed in the full 200° ultra-widefield retinal region as well as four standardized peripapillary quadrants (superior, inferior, nasal and temporal). The peripapillary annular measurement area was defined as the region extending from the optic disc margin to a fixed range of two disc diameters, and the four quadrants were divided by 45° axes centered on the optic disc. The core parameters included: fractal dimension, total vessel length, average branching angle, average caliber, arteriolar caliber, venular caliber, arteriole-to-venule ratio (AVR), average vessel tortuosity (separately for arteries and veins), and vessel density (e.g., vessel area density and vessel skeleton density). All parameter calculations were performed by automated algorithms, eliminating subjective bias and ensuring objectivity, accuracy, and repeatability.

### 2.6 Statistical analysis

All statistical analyses were performed using R software (version 4.4.1; R Foundation for Statistical Computing, Vienna, Austria). All tests were two-sided. Unless otherwise specified, a *P* < 0.05 was considered statistically significant before multiplicity correction, and an adjusted *P*-value or false discovery rate (FDR) < 0.05 was considered statistically significant after correction for multiple testing.

Continuous variables were summarized as mean ± standard deviation (SD) when approximately normally distributed and as median [interquartile range (IQR)] when the distribution was skewed. Categorical variables were summarized as counts and percentages. Baseline demographic and ocular characteristics were compared among the control, No-DR, and NPDR groups using one-way analysis of variance (ANOVA) or the Kruskal-Wallis test for continuous variables, as appropriate, and the chi-square test or Fisher’s exact test for categorical variables, as appropriate.

Multiparameter logistic regression models were built for four binary comparisons, using vascular parameters significant after age- and sex-adjusted FDR correction. Performance was assessed via participant-stratified 5-fold cross-validation with in-fold feature selection to avoid information leakage. ROC-AUC, optimal cutoff (Youden index) and diagnostic metrics were derived from out-of-fold predictions.

## 3 Results

### 3.1 Participant characteristics

A total of 237 participants were included in the analysis, comprising 63 control subjects, 101 patients with type 2 diabetes without diabetic retinopathy, and 73 patients with NPDR. At the eye level, 103, 201, and 132 eyes were available in the control, No-DR, and NPDR groups, respectively. Baseline demographic and ocular characteristics are summarized in Table 1.

**TABLE 1:** Baseline demographic and ocular characteristics of the study participants.

Characteristic	Control	No-DR	NPDR
Participants, n	63	101	73
Eyes, n	103	201	132
Age, years	70.35 ± 10.49	59.20 ± 14.27	62.73 ± 11.69
Female sex, n (%)	44 (69.8%)	47 (46.5%)	25 (34.2%)
Visual acuity	0.88 ± 0.16	0.83 ± 0.18	0.74 ± 0.23
Intraocular pressure, mmHg	15.59 ± 2.56	16.89 ± 2.54	16.76 ± 2.71

Values are n, n (%), or mean ± SD. *P*-values were calculated using one-way ANOVA for continuous variables and chi-square test for sex distribution. No-DR, diabetes without diabetic retinopathy; NPDR, non-proliferative diabetic retinopathy.

Age differed significantly among the three groups, with the control group being older on average (70.35 ± 10.49 years) than the No-DR (59.20 ± 14.27 years) and NPDR groups (62.73 ± 11.69 years). The proportion of female participants was also higher in the control group (69.8%) than in the No-DR (46.5%) and NPDR groups (34.2%). Therefore, subsequent vascular-parameter comparisons were adjusted for age and sex.

### 3.2 Age- and sex-adjusted differences in AI-derived retinal vascular parameters

After values coded as -1 in the AI export were treated as missing, parameters with insufficient valid observations were excluded from inferential interpretation. Age- and sex-adjusted mixed-effects models were then fitted for retained vascular parameters, followed by Benjamini-Hochberg FDR correction. After deduplication of parameters with identical displayed statistics, 38 vascular-parameter rows remained significant after FDR correction and are reported in Table 2. For Table 2 presentation, each parameter is displayed using a consistent descriptive format across the three groups.

**TABLE 2:** Retinal vascular parameters remaining significant after age/sex adjustment and FDR correction.

Category	Parameter	N	Control	No-DR	NPDR	Adjusted *P*	FDR *P*	Bonf. *P*	Effect
Vessel density	1-PD to 3-PD annular region vessel density	411	0.079 (0.035–0.109)	0.105 (0.089–0.122)	0.097 (0.076–0.113)	< 0.001	<0.001	0.002	Epsilon2 = 0.072
	1-cm circular region vessel density	411	0.008 (0.004–0.012)	0.012 (0.009–0.016)	0.010 (0.007–0.013)	< 0.001	<0.001	< 0.001	Epsilon2 = 0.137
	13.5-mm circular region vessel density	411	0.002 (0.001–0.003)	0.003 (0.002–0.004)	0.002 (0.002–0.003)	< 0.001	<0.001	< 0.001	Epsilon2 = 0.132
	3-PD circular region vessel density	411	0.086 (0.038–0.116)	0.113 (0.096–0.128)	0.103 (0.081–0.122)	< 0.001	<0.001	0.001	Epsilon2 = 0.070
	3-PD to 9-mm annular region vessel density	411	0.002 (0.001–0.004)	0.004 (0.003–0.006)	0.003 (0.002–0.004)	< 0.001	<0.001	< 0.001	Epsilon2 = 0.130
	9-mm circular region vessel density	411	0.004 (0.002–0.005)	0.005 (0.004–0.007)	0.004 (0.003–0.005)	< 0.001	<0.001	< 0.001	Epsilon2 = 0.134
	Whole-field vessel density (WFI)	427	0.017 (0.008–0.023)	0.026 (0.020–0.032)	0.021 (0.015–0.026)	< 0.001	<0.001	< 0.001	Epsilon2 = 0.164
	Inferonasal 1-cm annular region vessel density	405	0.004 (0.001–0.007)	0.007 (0.004–0.010)	0.005 (0.003–0.007)	< 0.001	<0.001	0.007	Epsilon2 = 0.085
	Inferotemporal 1-PD to 3-PD annular region vessel density	409	0.090 (0.043–0.121)	0.119 (0.095–0.135)	0.111 (0.076–0.131)	< 0.001	<0.001	0.008	Epsilon2 = 0.058
	Inferotemporal 1-cm annular region vessel density	409	0.006 (0.004–0.010)	0.011 (0.008–0.013)	0.009 (0.007–0.011)	< 0.001	<0.001	< 0.001	Epsilon2 = 0.102
	Inferotemporal 13.5-mm circular region vessel density	207	0.001 ± 0.001	0.002 ± 0.001	0.002 ± 0.001	< 0.001	<0.001	0.024	Eta2 = 0.101
	Inferotemporal 3-PD circular region vessel density	409	0.090 (0.043–0.127)	0.123 (0.098–0.137)	0.110 (0.076–0.135)	< 0.001	<0.001	0.014	Epsilon2 = 0.054
	Inferotemporal 9-mm circular region vessel density	207	0.002 ± 0.001	0.004 ± 0.001	0.003 ± 0.001	< 0.001	<0.001	0.022	Eta2 = 0.103
	Superonasal 1-PD to 3-PD annular region vessel density	408	0.071 (0.036–0.109)	0.099 (0.075–0.132)	0.088 (0.058–0.121)	0.002	0.006	0.245	Epsilon2 = 0.055
	Superonasal 1-cm annular region vessel density	407	0.010 (0.003–0.015)	0.014 (0.011–0.020)	0.012 (0.007–0.016)	< 0.001	<0.001	< 0.001	Epsilon2 = 0.092
	Superonasal 3-PD circular region vessel density	408	0.080 (0.039–0.124)	0.113 (0.087–0.147)	0.100 (0.071–0.136)	0.002	0.006	0.240	Epsilon2 = 0.053
	Superonasal 9-/13.5-mm circular region vessel density	206	0.010 (0.003–0.020)	0.018 (0.013–0.026)	0.013 (0.007–0.019)	< 0.001	<0.001	0.001	Epsilon2 = 0.116
	Superotemporal 1-PD to 3-PD annular region vessel density	409	0.099 (0.059–0.124)	0.117 (0.103–0.137)	0.119 (0.100–0.134)	< 0.001	<0.001	< 0.001	Epsilon2 = 0.054
	Superotemporal 1-cm annular region vessel density	409	0.013 ± 0.007	0.020 ± 0.007	0.016 ± 0.007	< 0.001	<0.001	< 0.001	Eta2 = 0.143
	Superotemporal 3-PD circular region vessel density	409	0.098 (0.062–0.129)	0.120 (0.107–0.141)	0.120 (0.101–0.137)	< 0.001	<0.001	< 0.001	Epsilon2 = 0.058
	Superotemporal 9-mm circular region vessel density	409	0.006 (0.002–0.016)	0.008 (0.005–0.023)	0.007 (0.004–0.019)	0.003	0.012	0.480	Epsilon2 = 0.045
Fractal dimension	3-PD circular region vessel fractal dimension	411	1.132 (1.011–1.207)	1.185 (1.133–1.225)	1.177 (1.124–1.215)	< 0.001	0.002	0.057	Epsilon2 = 0.034
	3-PD to 9-mm annular region vessel fractal dimension	411	1.215 (1.090–1.308)	1.333 (1.272–1.387)	1.281 (1.193–1.334)	< 0.001	<0.001	< 0.001	Epsilon2 = 0.151
	9-/13.5-mm circular region vessel fractal dimension	411	1.287 (1.164–1.359)	1.380 (1.328–1.420)	1.329 (1.271–1.374)	< 0.001	<0.001	< 0.001	Epsilon2 = 0.158
	Arterial fractal dimension	411	1.287 (1.164–1.359)	1.380 (1.328–1.420)	1.329 (1.271–1.374)	< 0.001	<0.001	< 0.001	Epsilon2 = 0.158
	Inferotemporal 6–9-mm annular region vessel fractal dimension	396	0.888 (0.785–0.975)	0.937 (0.883–0.997)	0.925 (0.839–0.985)	0.011	0.036	1.000	Epsilon2 = 0.035
	Inferotemporal 9-/13.5-mm circular region vessel fractal dimension	207	1.218 (1.103–1.291)	1.297 (1.256–1.345)	1.254 (1.196–1.304)	< 0.001	<0.001	0.019	Epsilon2 = 0.108
	Superonasal 3-PD circular region vessel fractal dimension	399	0.922 (0.837–1.006)	0.979 (0.922–1.031)	0.957 (0.881–1.015)	0.002	0.008	0.297	Epsilon2 = 0.036
	Superonasal 9-/13.5-mm circular region vessel fractal dimension	201	1.080 (0.910–1.196)	1.174 (1.094–1.233)	1.100 (1.001–1.176)	< 0.001	<0.001	0.006	Epsilon2 = 0.109
	Superotemporal 1-PD to 3-PD annular region vessel fractal dimension	411	1.099 (0.985–1.176)	1.156 (1.108–1.196)	1.151 (1.098–1.182)	< 0.001	<0.001	0.028	Epsilon2 = 0.039
	Superotemporal 9-/13.5-mm circular region vessel fractal dimension	408	1.188 (1.073–1.265)	1.280 (1.221–1.347)	1.232 (1.177–1.291)	< 0.001	<0.001	< 0.001	Epsilon2 = 0.119
Vessel caliber	1-cm circular region mean vessel diameter	411	11.329 (10.953–12.064)	11.158 (10.834–11.503)	11.389 (11.009–11.842)	< 0.001	0.001	0.042	Epsilon2 = 0.035
	3-PD circular region mean vessel diameter	406	12.243 (11.295–13.650)	12.040 (11.204–12.802)	12.491 (11.400–13.856)	0.008	0.028	1.000	Epsilon2 = 0.014
	3-PD to 9-mm annular region mean vessel diameter	410	11.081 (10.797–11.667)	11.039 (10.693–11.309)	11.193 (10.828–11.606)	0.007	0.024	1.000	Epsilon2 = 0.022
	9-/13.5-mm circular region mean vessel diameter	411	11.329 (10.953–12.064)	11.158 (10.834–11.503)	11.389 (11.009–11.842)	< 0.001	0.001	0.042	Epsilon2 = 0.035
	Whole-field mean vessel diameter (um)	427	12.743 (11.958–13.601)	12.163 (11.701–12.587)	12.420 (11.930–12.921)	< 0.001	<0.001	< 0.001	Epsilon2 = 0.060
	Superotemporal 9-/13.5-mm circular region mean vessel diameter	408	11.294 (10.701–12.190)	11.111 (10.732–11.446)	11.374 (10.772–11.997)	0.004	0.014	0.580	Epsilon2 = 0.023
Vessel length	Total vessel length (mm)	427	227.783 (208.721–264.487)	219.032 (200.853–237.408)	214.013 (190.687–235.634)	< 0.001	<0.001	0.001	Epsilon2 = 0.032

Values are presented row-wise as mean ± SD when all three groups were approximately normally distributed and as median (IQR) when any group showed non-normal distribution. Thus, the same descriptive format is used across Control, No-DR, and NPDR for each parameter. Adjusted P denotes the age- and sex-adjusted overall group-effect *P*-value from a linear mixed-effects model with subject as a random intercept. FDR P was calculated using the Benjamini-Hochberg procedure across vascular parameters; Bonf. P denotes Bonferroni-adjusted *P*-value.

Whole-field vessel density differed strongly among groups (Control, 0.017 (0.008–0.023); No-DR, 0.026 (0.020–0.032); NPDR, 0.021 (0.015–0.026); adjusted FDR P < 0.001). The No-DR group showed the highest whole-field vessel density, followed by NPDR and controls. In contrast, whole-field mean vessel diameter was lower in the No-DR group than in controls, with intermediate values in NPDR (Control, 12.743 (11.958–13.601); No-DR, 12.163 (11.701–12.587); NPDR, 12.420 (11.930–12.921); adjusted FDR P < 0.001). Total vessel length also differed among groups (Control, 227.783 (208.721–264.487); No-DR, 219.032 (200.853–237.408); NPDR, 214.013 (190.687–235.634); adjusted FDR P < 0.001).

**FIGURE 2:**
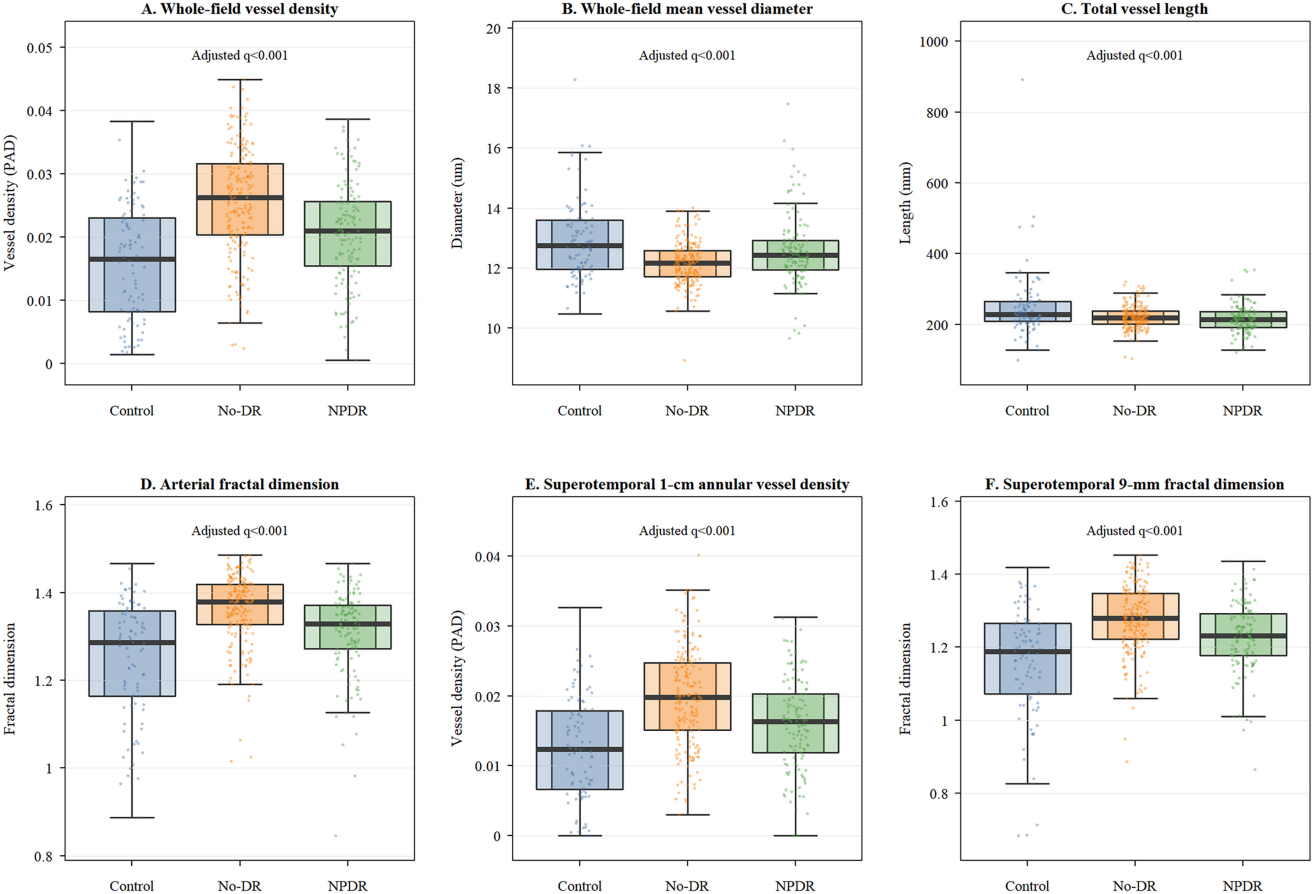
Representative distributions of key AI-derived retinal vascular biomarkers across the three study cohorts. To improve readability, the main figure was restricted to representative parameters that passed valid-sample-size quality control and remained significant after age/sex adjustment and Benjamini-Hochberg FDR correction. **(A)** Whole-field vessel density, **(B)** whole-field mean vessel diameter, **(C)** total vessel length, **(D)** arterial fractal dimension, **(E)** superotemporal 1-cm annular vessel density, and **(F)** superotemporal 9-mm circular fractal dimension. Boxes represent the interquartile range with median lines; whiskers indicate 1.5 times the interquartile range; dots represent individual eyes. Control, healthy non-diabetic subjects; No-DR, patients with type 2 diabetes without clinical diabetic retinopathy; NPDR, patients with early non-proliferative diabetic retinopathy; PAD, pixel area density; WFI, wide-field image; FDR, false discovery rate.

**FIGURE 3:**
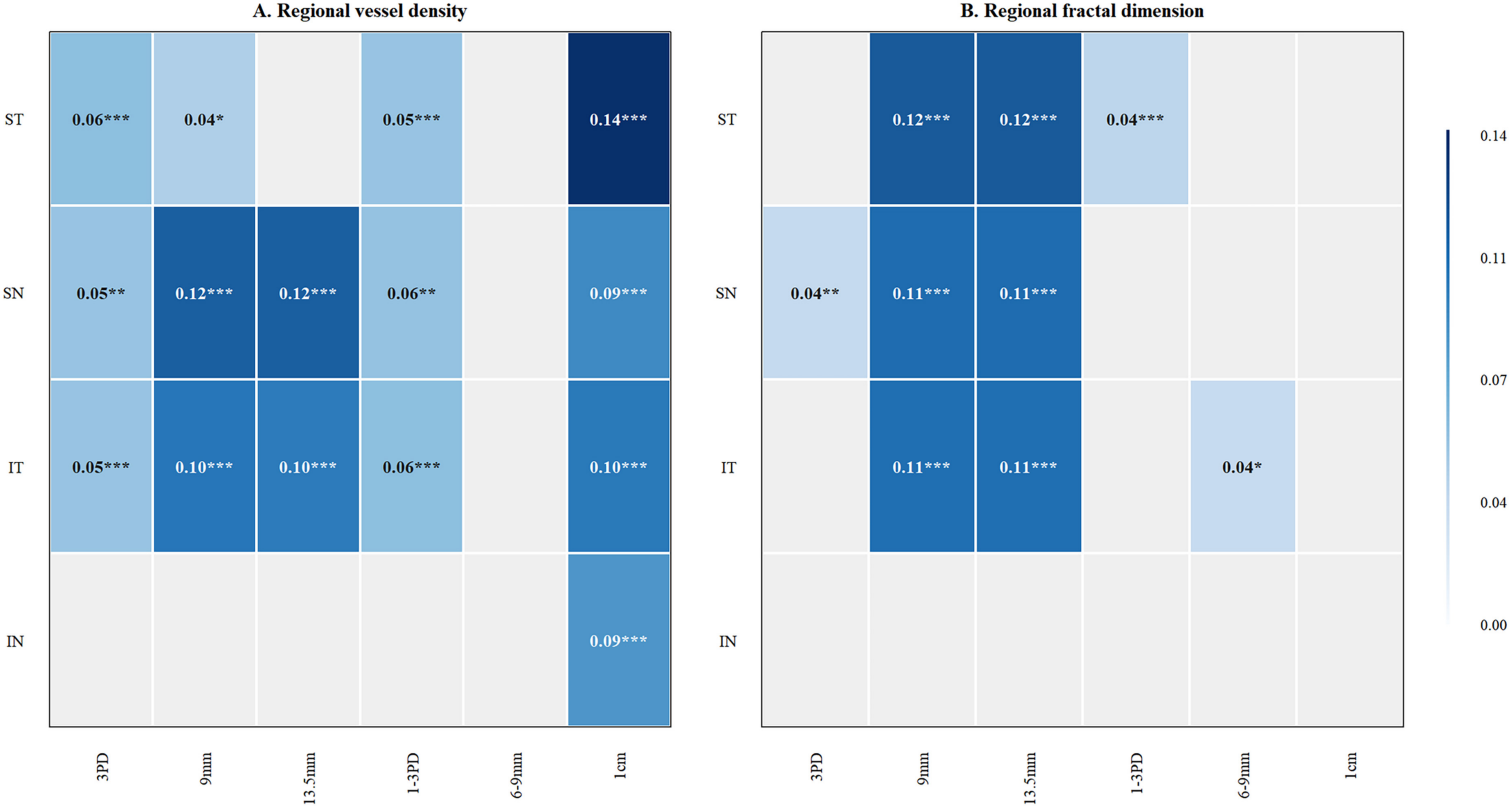
Regional summary heatmaps of retinal vascular alterations after age/sex adjustment and multiple-testing correction. Heatmaps summarize quadrant-specific regional vessel density and fractal-dimension parameters using effect size for parameters with Benjamini-Hochberg FDR-adjusted *P* < 0.05. Gray cells indicate parameters that were not significant after FDR correction or were not available for that region. Cell values denote effect size; *, **, and *** indicate adjusted *P* < 0.05, adjusted *P* < 0.01, and adjusted *P* < 0.001, respectively. IN, inferonasal; IT, inferotemporal; SN, superonasal; ST, superotemporal; FDR, false discovery rate.

Fractal-dimension parameters showed robust group differences after adjustment. Arterial fractal dimension was higher in the No-DR group [1.380 (1.328–1.420)] than in controls [1.287 (1.164–1.359)], with NPDR showing intermediate values [1.329 (1.271–1.374); adjusted FDR *P* < 0.001]. Consistent regional signals were observed in 9-/13.5-mm circular fractal-dimension metrics and in the 3-PD to 9-mm annular region. Representative distributions are shown in Figure 2, and the broader regional pattern is summarized in Figure 3.

### 3.3 Regional patterns of retinal vascular alteration

Regional heatmap analysis demonstrated that significant vessel-density alterations were most prominent in the superotemporal, superonasal, and inferotemporal regions, particularly in the 1-cm annular, 9-mm circular, and 13.5-mm circular regions. The strongest regional density effect was observed in the superotemporal 1-cm annular region (effect size 0.143, adjusted FDR *P* < 0.001). Regional fractal-dimension signals were concentrated mainly in the superior and temporal quadrants, especially in the 9-mm and 13.5-mm circular regions. Grey cells in Figure 3 indicate parameters that were not significant after FDR correction or were not available for that region.

### 3.4 Multiparameter diagnostic model performance

**FIGURE 4:**
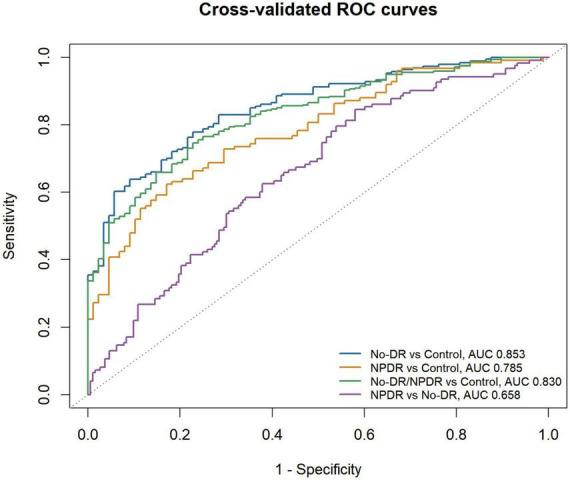
Cross-validated receiver operating characteristic curves of the multiparameter diagnostic models. Curves were generated from out-of-fold predicted probabilities in 5-fold subject-level cross-validation. The combined vascular-feature models showed good discrimination for No-DR versus control, NPDR versus control, and any diabetes/NPDR versus control, whereas discrimination between NPDR and No-DR was more modest. AUC, area under the receiver operating characteristic curve; No-DR, diabetes without diabetic retinopathy; NPDR, non-proliferative diabetic retinopathy.

To evaluate whether combined vascular parameters could support individualized classification, multiparameter diagnostic models were constructed and evaluated using 5-fold subject-level cross-validation. Model performance is summarized in Table 3 and Figure 4.

**TABLE 3:** Cross-validated performance of the multiparameter diagnostic models.

Model	Eyes	Subjects	AUC (95% CI)	Cutoff	Sensitivity	Specificity	PPV	NPV	Accuracy
No-DR vs. Control	282	152	0.853 (0.807–0.893)	0.680	0.778	0.773	0.883	0.613	0.777
NPDR vs. Control	213	117	0.785 (0.720–0.841)	0.695	0.624	0.830	0.839	0.608	0.709
No-DR/NPDR vs. Control	408	218	0.830 (0.788–0.873)	0.799	0.747	0.773	0.923	0.456	0.752
NPDR vs. No-DR	316	166	0.658 (0.596–0.720)	0.274	0.846	0.420	0.481	0.810	0.585

Performance metrics were estimated using out-of-fold predictions from 5-fold subject-level cross-validation. Cutoff denotes the Youden-index optimal predicted-probability threshold. AUC, area under the receiver operating characteristic curve; PPV, positive predictive value; NPV, negative predictive value.

The No-DR versus control model achieved an AUC of 0.853 (95% CI, 0.807–0.893), with sensitivity 0.778 and specificity 0.773. The NPDR versus control model achieved an AUC of 0.785 (95% CI, 0.720–0.841). When No-DR and NPDR eyes were combined and compared with controls, the model achieved an AUC of 0.830 (95% CI, 0.788–0.873). Discrimination between NPDR and No-DR was more modest, with an AUC of 0.658 (95% CI, 0.596–0.720).

The features most consistently selected across cross-validation folds included whole-field vessel density, superotemporal 1-cm annular vessel density, superotemporal 9-mm circular fractal dimension, total vessel length, and selected regional density metrics (Table 4). Decision curve analysis showed positive net benefit over a range of threshold probabilities for the main control-comparison models and is provided as Supplementary Figure 1.

**TABLE 4:** Vascular features most consistently selected in cross-validation.

Model	Selected feature	Selected folds	Frequency
No-DR vs. Control	Superotemporal 1-cm annular region vessel density	5	1.0
No-DR vs. Control	Superotemporal 9-mm circular region vessel fractal dimension	5	1.0
No-DR vs. Control	Whole-field vessel density (WFI)	5	1.0
No-DR vs. Control	Superonasal 1-cm annular region vessel density	4	0.8
NPDR vs. Control	Superotemporal 1-cm annular region vessel density	4	0.8
NPDR vs. Control	Superotemporal 1-PD to 3-PD annular region vessel density	4	0.8
NPDR vs. Control	Superotemporal 9-mm circular region vessel fractal dimension	4	0.8
NPDR vs. Control	Whole-field vessel density (WFI)	4	0.8
NPDR vs. Control	Total vessel length (mm)	4	0.8
No-DR/NPDR vs. Control	Superotemporal 1-cm annular region vessel density	5	1.0
No-DR/NPDR vs. Control	Superotemporal 9-mm circular region vessel fractal dimension	5	1.0
No-DR/NPDR vs. Control	Whole-field vessel density (WFI)	5	1.0
No-DR/NPDR vs. Control	1-PD to 3-PD annular region vessel density	4	0.8
NPDR vs. No-DR	Superonasal 1-cm annular region vessel density	5	1.0
NPDR vs. No-DR	Inferonasal 1-cm annular region vessel density	5	1.0
NPDR vs. No-DR	Superotemporal 9-mm circular region vessel fractal dimension	5	1.0
NPDR vs. No-DR	Superotemporal 1-cm annular region vessel density	4	0.8
NPDR vs. No-DR	Arterial fractal dimension	4	0.8

Only features selected in at least 4 of 5 cross-validation folds are shown. Full feature-selection results are provided in the diagnostic-model output workbook.

## 4 Discussion

Diabetic retinopathy (DR) remains the leading cause of preventable blindness in working-age populations worldwide (2, 19), and early detection and intervention are critical for preserving visual function. However, conventional clinical examination can only detect DR when structural lesions such as microaneurysms or hemorrhages have already appeared, missing the optimal window for early intervention (20). In this study, we used an artificial intelligence (AI)-based retinal image analysis system to quantitatively evaluate multiple retinal vascular morphological parameters in patients with no clinical signs of DR (no-DR) and early non-proliferative diabetic retinopathy (NPDR), as well as healthy controls. Our results demonstrated that significant retinal vascular alterations occur even in the preclinical stage of DR, before the appearance of clinically visible lesions (21). These findings provide new insights into the early pathogenesis of DR and highlight the potential of AI-based quantitative vascular analysis as a valuable tool for early DR screening (22).

The most consistent finding was the alteration of vessel-density-related parameters. Whole-field vessel density was highest in the No-DR group, intermediate in the NPDR group, and lowest in controls. Similar patterns were observed across multiple annular and circular regions, particularly in the 1-cm, 9-mm, and 13.5-mm zones. This non-linear pattern suggests that early diabetes may be accompanied by vascular redistribution or compensatory microvascular remodeling, whereas progression to clinically visible NPDR may involve partial loss of this compensatory pattern. Therefore, retinal vascular density should not be interpreted simply as increasing or decreasing with disease severity; rather, it may reflect a dynamic balance between early vascular adaptation, altered perfusion demand, and later microvascular damage. Consistent with our findings, previous optical coherence tomography angiography (OCTA) studies have also documented increased macular capillary density in diabetic patients without clinically apparent retinopathy, supporting the presence of subclinical microvascular proliferation prior to visible DR lesions (23, 24). This early vascular remodeling is thought to be driven by chronic retinal hypoxia and elevated metabolic demand under hyperglycemic conditions, which trigger compensatory angiogenic signaling pathways (25). A recent meta-analysis of OCTA studies has consistently confirmed that peripapillary and macular vessel density are significantly reduced in diabetic patients without clinical retinopathy, providing robust evidence that microvascular impairment precedes the onset of clinically diagnosable DR (26).

Fractal dimension is a quantitative measure of the complexity and branching pattern of the retinal vascular network (27, 28). Fractal-dimension metrics also showed robust group differences, especially in arterial, 9-/13.5-mm circular, and 3-PD to 9-mm annular regions. Higher fractal dimension in the No-DR group suggests increased geometric complexity of the retinal vascular network at an early diabetic stage. The intermediate values observed in NPDR may indicate that vascular complexity increases during early remodeling but becomes attenuated as structural damage progresses. Together with the vessel-density findings, this supports the concept that diabetes-related retinal microvascular changes begin before clinically defined retinopathy and involve both vascular occupancy and network architecture (29). A 6-year cohort study demonstrated that several baseline retinal vascular geometric parameters independently predict the incidence and progression of diabetic retinopathy, and they can enhance risk prediction when used alongside conventional clinical factors (30). These findings are consistent with the concept of early compensatory angiogenesis in diabetes (31).

In the early stages of diabetes, retinal hypoxia and increased metabolic demand trigger a compensatory angiogenic response to improve tissue perfusion. This leads to the formation of new small capillaries, which increases the overall vascular density and complexity of the network, resulting in higher fractal dimension values. However, this compensatory response is ultimately maladaptive, as these new vessels are structurally and functionally abnormal, and eventually contribute to the development of more advanced DR. Interestingly, we observed that both fractal dimension and vessel density were higher in the no-DR group than in the NPDR group. These findings align with the concept of early compensatory angiogenesis in diabetes, which is central to the biphasic vascular change theory of diabetic retinopathy first proposed by Klein et al. This theory states that diabetic retinal vascular changes follow a two-stage pattern: the early stage (no-DR to mild NPDR) is dominated by compensatory angiogenesis and vasodilation, manifesting as increased vascular density and fractal dimension; the late stage (moderate NPDR onwards) is characterized by vascular loss and nonperfusion, leading to a subsequent decline in both parameters (32).

Regional heatmap analysis further demonstrated that these alterations were spatially heterogeneous rather than uniformly distributed across the retina. Significant changes clustered mainly in the superotemporal, superonasal, and inferotemporal regions, with the superotemporal 1-cm annular vessel density showing one of the strongest effects. This regional predominance suggests that quadrant-specific and peripheral retinal measurements may provide additional information beyond global indices. Wide-field imaging may therefore be particularly useful for detecting early diabetic vascular remodeling that could be missed by analyses restricted to the posterior pole. This spatial heterogeneity is consistent with previous ultra-widefield angiography studies, which demonstrated that a substantial proportion of diabetic retinal ischemia and microvascular lesions occur outside the traditional Early Treatment Diabetic Retinopathy Study (ETDRS) 7-field area, particularly in the temporal and superior peripheral retina (33, 34). Conventional posterior pole-only imaging would therefore underestimate the full extent of early diabetic vascular damage, supporting the added value of ultra-widefield imaging in early DR assessment (35, 36).

Vessel caliber and total vessel length provided complementary information to vessel density. Whole-field mean vessel diameter was lower in the No-DR group than in controls, with intermediate values in NPDR, while total vessel length showed a decreasing trend across controls, No-DR, and NPDR. These findings suggest that early diabetic retinal vascular remodeling is not characterized simply by vascular expansion, but by coordinated changes in vessel occupancy, caliber, and network extent. Importantly, vessel density in this study was calculated as pixel area density within the analyzable retinal region, whereas total vessel length and mean vessel diameter were derived from skeletonized vessel extent and local caliber, respectively. Therefore, discordant patterns among these parameters should be interpreted as evidence that they capture distinct aspects of vascular morphology rather than as contradictory findings. Retrospective analysis with ultrawide field imaging indicates that outer retinal arteriolar narrowing relates to greater nonperfusion, more severe diabetic retinopathy and peripheral lesions, unlike vascular caliber in the inner zone (37). A 3-year prospective study of 904 adolescents with type 1 diabetes demonstrated that enlarged retinal vessel calibers in the area over 2 disc diameters from the optic disc independently predicted moderate diabetic retinopathy, while peripapillary vessel calibers showed no such association (38). Consistent with previous findings (39), our study also demonstrated retinal venular widening in the NPDR group. Retinal venular widening has been proven to correlate with the development and progression of diabetic retinopathy.

The findings of our study have important clinical implications. First, they demonstrate that significant retinal vascular alterations occur long before the appearance of clinically visible DR lesions. This suggests that quantitative analysis of retinal vascular parameters can be used to identify patients at high risk of developing DR, allowing for earlier intervention and better visual outcomes. Second, the use of AI technology enables automated, rapid, and accurate measurement of these parameters (40), overcoming the limitations of manual measurement, which is time-consuming, subjective, and prone to inter-observer variability. This makes it feasible to implement large-scale population-based screening for early DR using AI-based retinal image analysis, particularly in resource-limited settings where access to ophthalmologists is limited.

Several limitations should be acknowledged. First, this was a retrospective single-center study, and external validation is required before clinical application. Second, although age and sex were adjusted for in the main analyses, residual confounding cannot be fully excluded. Third, the cross-sectional design prevents direct inference about temporal progression from No-DR to NPDR. Fourth, some AI-derived parameters, such as venous tortuosity, had sparse valid observations and were therefore unsuitable for primary interpretation. Finally, model performance was internally cross-validated but still requires validation in independent cohorts and across different imaging systems.

## 5 Conclusion

In conclusion, AI-derived wide-field retinal vascular metrics reveal early, spatially heterogeneous microvascular remodeling in type 2 diabetes, even before clinically apparent diabetic retinopathy. Vessel density, fractal dimension, vessel caliber, and vessel length provide complementary information about diabetic retinal vascular change. The diagnostic modeling results further support the potential of multiparameter retinal vascular analysis as an adjunctive tool for early detection and risk stratification, although external validation and longitudinal studies are needed before routine clinical use.

## Data Availability

The original contributions presented in this study are included in the article/Supplementary material, further inquiries can be directed to the corresponding author.
