# Supplementary material for: Artificial intelligence–based analysis of retinal vascular changes in the preclinical and early stages of diabetic retinopathy using ultra-widefield fundus imaging: an observational cross-sectional study
Source: Front Med (Lausanne). 2026 Jul 14;13:1906162. doi: 10.3389/fmed.2026.1906162 (PMC13407527; doi:10.3389/fmed.2026.1906162)
Supplement: Supplementary file 1 [file Image_1.pdf]

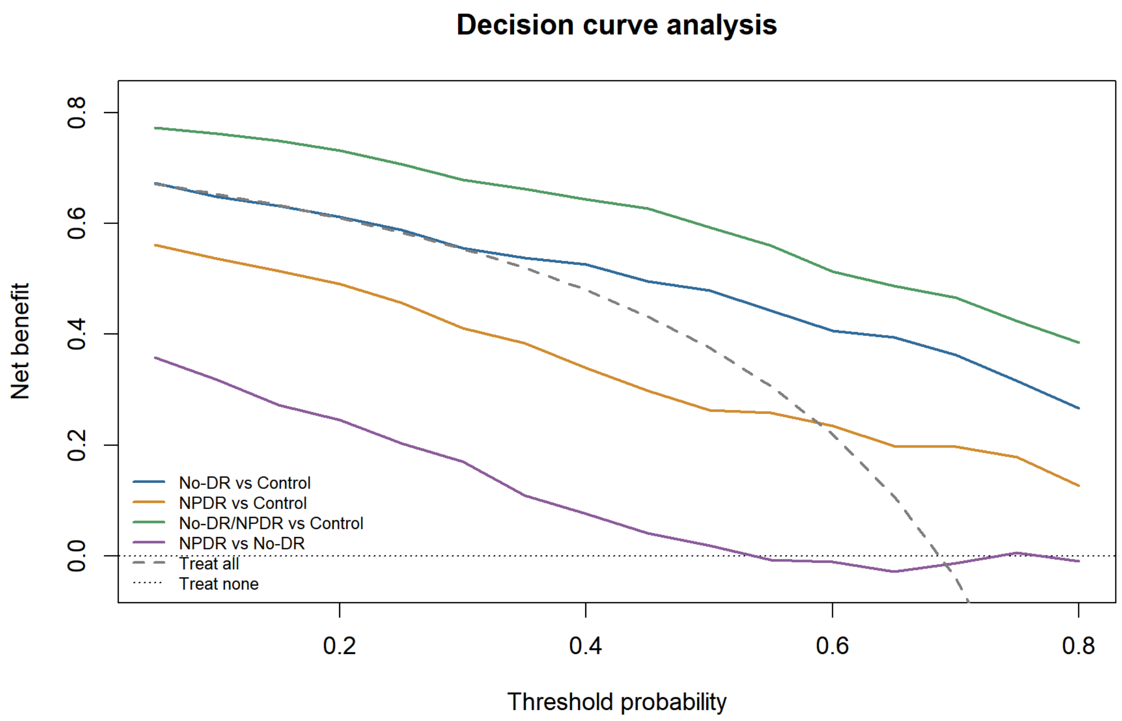

Supplementary Figure S1. Decision curve analysis of the multiparameter diagnostic models. Net benefit was plotted across clinically relevant threshold probabilities and compared with treat-all and treat-none reference strategies. This figure is recommended for supplementary material because it supports clinical utility assessment without overloading the main figure set.
